# Reestablishing the Function and Esthetics in Traumatized Permanent Teeth with Large Apical Lesion

**DOI:** 10.1155/2016/3830813

**Published:** 2016-12-18

**Authors:** Alexandra Rubin Cocco, Ângelo Niemczewski Bobrowski, Rudimar Antônio Baldissera, Luiz Fernando Machado Silveira, Josué Martos

**Affiliations:** ^1^Department of Operative Dentistry, School of Dentistry, Federal University of Pelotas, Pelotas, RS, Brazil; ^2^Department of Oral and Maxillofacial Surgery, School of Dentistry, Federal University of Pelotas, Pelotas, RS, Brazil; ^3^Department of Semiology and Clinics, Faculty of Dentistry, University Federal of Pelotas, Pelotas, RS, Brazil

## Abstract

Dental trauma is a challenge for dental integrity and can lead to pulp necrosis. The clinical case reports the diagnosis of a maxillary right central incisor traumatized and its multidisciplinary treatment. Calcium hydroxide material was used to perform the processing apexification. An apical surgery was carried out to remove the apical periodontitis and to return the aesthetics to the patient; internal and external tooth whitening in maxillary right central incisor was performed. We conclude that surgery associated with the root filling in the central incisor led to a successful completion. Moreover, it is of utmost importance to demonstrate the interaction between the various areas of dentistry.

## 1. Introduction

The eruption of permanent teeth occurs between 6 and 16 years [1], and any factor that interferes in this physiological process can interfere with root development [2]. A classic example of this interference is the occurrence of dental trauma. Most of these injuries happen before the complete formation of the dental root, predisposing as an immediate consequence a pulp inflammation or pulp necrosis [3]. In addition to the resulting inflammation the complete destruction of the Hertwig's epithelial root sheath may occur, causing an outage of root formation.

Moreover, consequences may be generated with incomplete root formation as such invasion of bacteria favors the formation of periapical lesions [4]. The presence of apical periodontitis will promote the formation of cysts in all cases and the conventional endodontic treatment alone will not be enough [5]. In these clinical situations, multidisciplinary interventions may be required.

In this context, the use of apexification technique has been recommended to induce a calcified root barrier to open apex or incomplete root apical development associate with pulp necrosis [6, 7]. Calcium hydroxide is the most common material used to induce the formation of apical hard tissue [8, 9], with success rates of 74–100% [1, 8, 10]. The mineral trioxide aggregate (MTA), due to showing favorable properties including biocompatibility [11–13], has also been used for the same purpose, showing similar results [10]. Other treatment recommended in cases of incomplete root formation is revascularization. A study performed revascularization/revitalization therapy in traumatized anterior teeth and it had a high clinical success rate in one-year follow-up [14].

The paper presents a case report of a patient with large periapical lesion that was treated by apical curettage followed by the apexification technique using calcium hydroxide medication and final root canal sealing with MTA.

## 2. Case Presentation 

A 21-year-old female patient sought dental care due to bulging in the palate region, as well as dissatisfaction with the color of your maxillary right central incisor.

The patient reported having suffered a facial trauma in an anterior-superior direction arising caused by a stone impact. The trauma resulted in a fractured maxillary right central incisor, in addition to a mucous fistula appearance in the region after some time. She highlighted a new history of trauma in the same region in the past three years of the first event.

**Figure 1:**
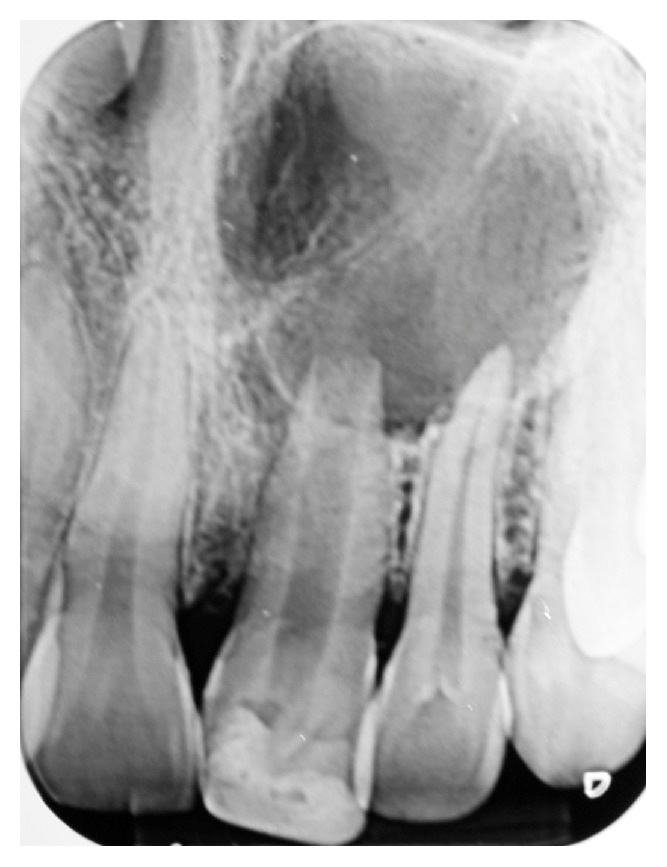
Initial radiograph of the maxillary right central incisor.

During clinical examination, a light mobility in teeth maxillary right central incisor (11) was verified, in addition to an expansion of about 2 cm^2^ of the palatal cortical bone and presenting hard consistency. Palpation in the palate with light pressure had drainage of large amounts of purulent secretion. Diagnostic evaluation showed negative response to pulp sensitivity tests in the upper right central incisor, but the adjacent teeth showed pulp normality. By radiographic examination a circumscribed lesion in the apical region of the maxillary right central incisor with presumed periapical cyst and an incomplete apical root formation was observed (Figure 1).

The clinical planning highlighted the need for surgical intervention in the apical region of the central incisor associated with endodontic therapy and later following a second stage of the treatment comprising the cosmetic/restorative procedures.

**Figure 2:**
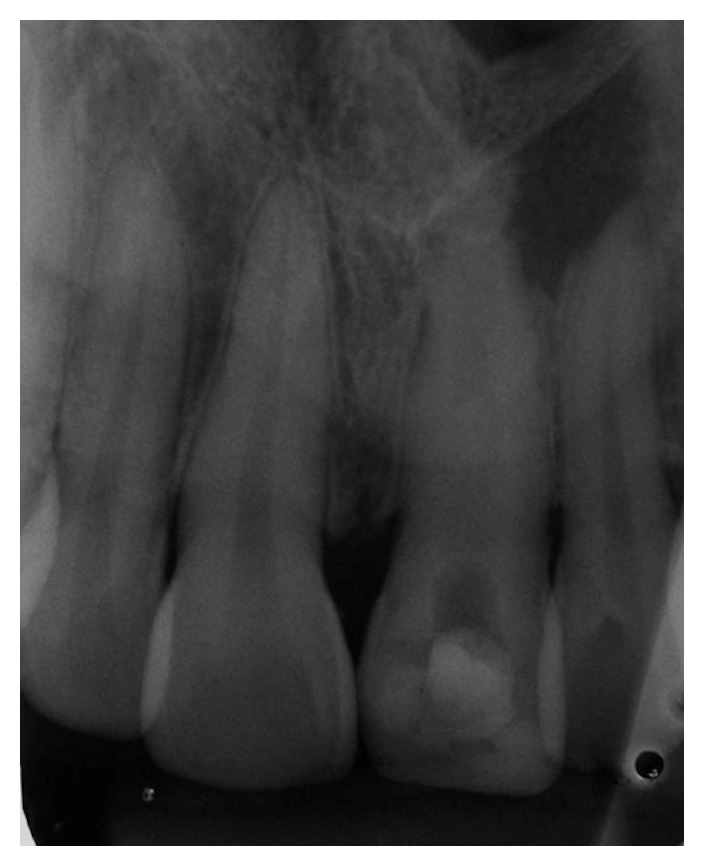
Intracanal medication of calcium hydroxide.

Endodontic treatment started with complete rubber dam isolation without dental clamps of tooth maxillary right central incisor (11) and endodontic access following copious irrigation of the pulp chamber and cervical third. The root canal was cleaned with endodontic K-files (Dentsply-Maillefer, Ballaigues, Switzerland) until the working length was reached, and it was copiously irrigated with sodium hypochlorite (NaOCl) at 2.5% alternated with 17% EDTA, aspirated, and dried with absorbent cones. After root canal preparation the application of calcium hydroxide paste was performed. Intracanal medication of calcium hydroxide (Callen, SS White, Rio de Janeiro, RJ, Brazil) was applied prior to the surgical procedure and sealed with glass ionomer restorative material (Figure 2).

**Figure 3:**
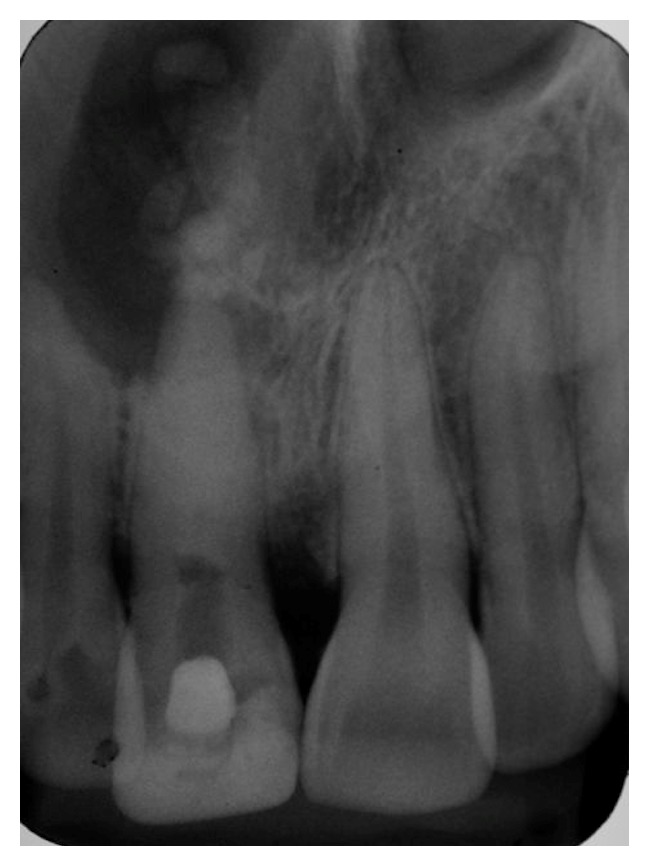
Postsurgical aspect.

After performing antisepsis, local anesthesia, and incision the mucoperiosteum flap displacement was done and osteotomy was carried out to allow access to the affected area. It was possible to perform the excision of the periapical process and a careful curettage of the apical area. The biopsy of the specimen identified dense fibrous connective tissue, exhibiting intense inflammatory infiltrate and diffuse lymphocytic, confirming the diagnosis of periapical cyst. After a week of paraendodontic surgery, the patient had no asymmetry of the alveolar mucosa in the palate as well as exudation. Radiographically, the operated area and the need of new root application of calcium hydroxide paste were evident (Figure 3).

**Figure 4:**
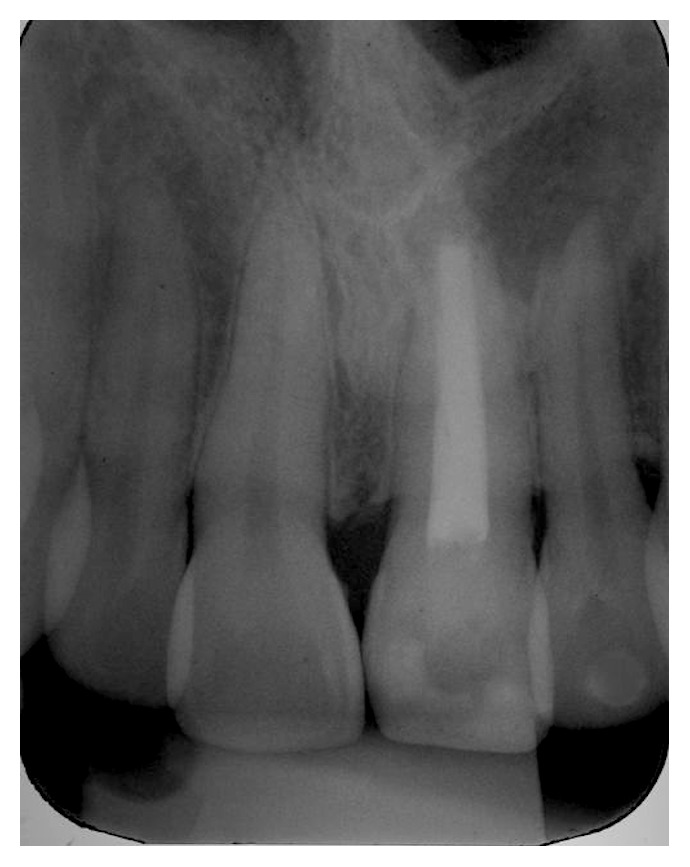
Root canal final filling with a MTA-based endodontic cement.

After six months, the operative procedures for permanent obturation of the root canal of the central incisor were started. After the removal of intracanal calcium hydroxide, the root canal final filling with cone rolled technique associated with a MTA-based endodontic cement was performed (MTA-Fillapex, Angelus, Londrina, PR, Brazil) (Figure 4).

**Figure 5:**
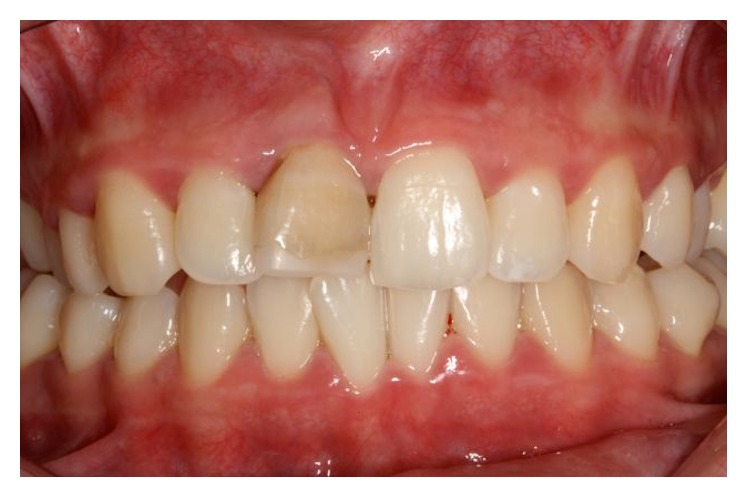
Crown darkening of maxillary right central incisor.

The crown darkening of tooth maxillary right central incisor (11) observed by the color scale (Vitapan 3D-Master, Vita Zahnfabrik GmbH, Germany) provided the use of dental office whitening technique (Figure 5). The clinical office whitening procedure began with the application of a light-cured gingival barrier at the cervical region of the tooth to be cleared to the protection of gingival tissue (Gingi Dam, Villevie, Dentalville, Joinville, Brazil). A twist-pen applicator with hydrogen peroxide at 35% was used (Mix One Supreme, Villevie, Dentalville, Joinville, Brazil) following the manufacturer's instructions. A layer of gel based on hydrogen peroxide (Mix One Supreme, Villevie, Dentalville, Joinville, SC, Brazil) was applied using external crown application only.

**Figure 6:**
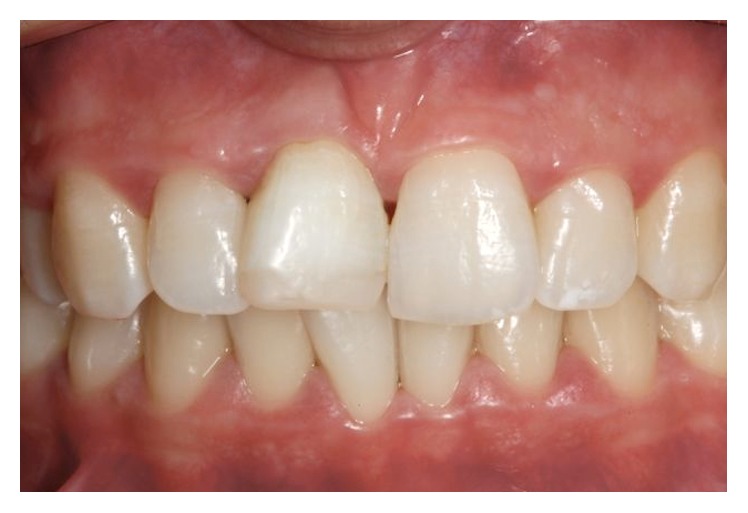
Final result after bleaching procedure.

Three applications of whitening product were performed during forty-five minutes in one unique clinical session, and, at the end of the proposed treatment, a satisfactory change was observed in the chromatic aspect in the color of the central incisor (Figure 6). The clinical and radiographic follow-up shows a satisfactory outcome for the dental specialties involved in this treatment.

## 3. Discussion

The multidisciplinary integration is very important for planning and executing a dental treatment. In this clinical report success with the approach can be seen through the interaction of distinct areas like surgical, endodontic, and operative dentistry.

After surgical removal of the lesion, the endodontic technique of apexification was performed. This technique consists in applying a biocompatible material into the root canal to establish an apical stop allowing the root filling in future to induce root formation and subsequent closing of the apical foramen. This is possible due to the deposit of mineralized hard tissue composed of osteocement, osteodentin, or bone or a combination of these three tissues at apical level [6, 7].

Currently, other technique has been widely used for immature teeth with nonvital pulp as the revascularized/revitalized teeth. This technique induces apexogenesis. It is new treatment modality that uses, instead of tissue replacement using artificial substitutes, tissue regeneration [1, 15]. However, this technique was not used in this study and due to the absence of well conducted long-term studies the technique raises several questions, among which is whether the technique serves as a permanent treatment or whether filling of the canal space is recommended. In our knowledge, there is only one study with one year follow-up [14]. It is necessary to have more studies in the long term.

However, apexification treatment has been a routine procedure to treat and preserve such teeth for many decades [15, 16]. The process of apexification forms an apical barrier, so that the subsequent condensation of the filling material canal can be adequately achieved [17]. Traditionally, the most common material used is calcium hydroxide, due to their biological stimulation capability, their osteogenic potential, and their antibacterial action [17–21]. These properties are related to the highly released and extremely reactive hydroxyl ions. These ions cause damage to the bacterial citoplasmatic membrane, denaturing their proteins and providing irreversible damage to their DNA [17–21].

Calcium hydroxide has been used successfully in the apical barrier formation in 74–100% of cases [8, 9]. Some studies show that 86% of treated teeth showed a survival rate between 5 and 13 years [22–24]. However, this material has been replaced by the MTA because it is necessary to do various changes, generating a time-consuming treatment, which can vary between 3 and 17 months [9].

For the root canal filling, sealer Fillapex MTA was used. This material has some satisfactory properties such as good seal and good apical barrier consistency forming hard tissue [11, 12]. In addition, it promotes the efficient apical seal in the dentin and cementum, facilitating biological repair and regeneration of the periodontal ligament.

Regarding the bleaching procedures, scientific evidence shows that free radicals of hydrogen peroxide not catalyzed for tooth whitening are those that can cause the phenomenon of external cervical root resorption due to the inflammatory process. The cementoenamel junction is the point of fragility of the tooth structure because it can expose the dentin. In an attempt to prevent the spread of bleaching products on the outer surface at the cementoenamel junction and prevent an inflammatory response in the surrounding periodontal tissues, a protection or a gingival barrier was used at the cervical level before applying the bleaching product [7].

In view of these considerations it is important to note that when the tooth has been traumatized and requires bleaching, the first choice should be to use external application [7]. As an added precaution, the bleaching treatment was not made using hydrogen peroxide associated with heat, and the hydrogen peroxide was only applied to the external enamel surface.

## 4. Conclusion

Through a multidisciplinary approach, it was possible to obtain a clinical success of the case presented by restoring the function and aesthetics of the patient.
